# Different risk factors for multiple and unifocal gliomas: a comparative study of radiological, pathological and clinical characteristics

**DOI:** 10.3389/fonc.2025.1531879

**Published:** 2025-05-27

**Authors:** Limei Feng, Xinyao Shi, Yuying Zang, Xuzhu Chen

**Affiliations:** ^1^ Department of Radiology, Beijing Tiantan Hospital, Capital Medical University, Beijing, China; ^2^ Department of Radiology, Capital Center for Children's Health, Capital Medical University, Beijing, China

**Keywords:** multicentric gliomas, multifocal gliomas, diagnosis, differentiation, prognosis

## Abstract

**Background:**

This retrospective study compared two types of gliomas and two subtypes of multiple gliomas.

**Methods:**

The clinical manifestations, magnetic resonance imaging (MRI) findings, pathological characteristics, and clinical outcomes of 188 patients with unifocal and 94 patients with multiple gliomas (59 with multifocal and 35 with multicentric gliomas) were analyzed.

**Results:**

Compared with patients with unifocal glioma, those with multiple gliomas were older (*P*=0.001) and more likely to be male (χ^2^ = 4.857, *P*=0.028). Patients with multiple gliomas had smaller extent of surgical resection (χ^2^ = 161.016, *P*<0.001) and a worse prognosis (χ^2^ = 43.733, *P*<0.001) than those with unifocal gliomas. Multiple gliomas were more likely to have a non-superficial location (χ^2^ = 51.758, *P*<0.001), obvious peritumoral oedema (χ^2^ = 9.688, *P*=0.008), intense enhancement (χ^2^ = 24.547, *P*<0.001), a higher WHO grade (*P*=0.001), a lower ratio of isocitrate dehydrogenase (IDH) mutation (χ^2^ = 51.770, *P*<0.001), and codeletion of 1p19q (χ^2^ = 8.637, *P*=0.003). Tumor location and IDH status were identified as independent risk factors for multiple gliomas (*P*<0.001 and *P*=0.003, respectively). Deep tumor location was found to be the only factor related to unfavorable overall survival (OS) in multiple gliomas. Patients with multifocal gliomas were more likely to be male than patients with multicentric gliomas (χ^2^ = 6.521, *P*=0.011). The locations of multifocal and multicentric gliomas were significantly different (*P*=0.048). WHO grade was identified as an independent prognostic factor (*P*=0.034) in patients with multicentric gliomas but not in those with multifocal gliomas.

**Conclusions:**

The demographic characteristics, extent of resection, radiological features, pathological features and prognostic factors differ between patients with multiple gliomas and those with unifocal gliomas. The clinical and radiological features differ between patients with different subtypes of multiple gliomas. Multiple gliomas located only in superficial regions are more likely to be multicentric gliomas and the prognosis is solely related to the WHO grades, providing valuable guidance for clinical treatment.

## 1 Introduction

Gliomas account for 22% of all primary brain and other central nervous system tumors (1). Adult diffuse glioma is the most common primary malignant tumor of the central nervous system (2). Gliomas are very heterogeneous (3, 4) and may include a single lesion (unifocal glioma) or multiple lesions. Multiple gliomas can be divided into clearly-defined multicentric and multifocal subtypes (5). Multifocal gliomas communicate through white matter fibers between two lesions, have multiple satellite foci adjacent to the main tumor, or have separate lesions abutting the ventricular system. Multicentric gliomas are isolated lesions in different lobes or hemispheres that are not characterized as multifocal gliomas (6).

Previous studies focused on the differentiation of multiple gliomas from other multiple brain lesions (7) and the comparison of multiple gliomas with unifocal gliomas (8, 9). Recent radiological studies have focused on white matter involvement sites, accurate segmentation of the lesions (10), and prognostic imaging markers (11, 12). However, these studies mainly focused on high-grade multiple gliomas and did not include low-grade multiple gliomas, and the two subtypes of multiple gliomas have rarely been compared in the literature. Therefore, this study compares the clinical, radiological, and pathological differences between unifocal and multiple gliomas in low-grade and high-grade gliomas. Furthermore, multifocal and multicentric gliomas are also compared in this study.

## 2 Materials and methods

The institutional review board of Beijing Tiantan Hospital, Capital Medical University approved this retrospective study.

### 2.1 Patient selection

A total of 955 consecutive adult patients with cerebral glioma (aged 18–87 years; mean age, 46.49 ± 13.55 years) were recruited for this study from the neuro-oncology database of our institution between January 2020 and December 2020. Patients ≥18 years old with cerebral gliomas and available surgical treatment data were included. Patients <18 years with uncertain pathology results, recurrent glioma, spinal cord glioma, missing surgical treatment data or preoperative magnetic resonance imaging (MRI) data, or preoperative MRI without fluid-attenuated inversion recovery (FLAIR) images were excluded from the study. A patient flowchart is shown in **Supplementary Figure 1**. The pathological grades of all recruited patients were readjusted according to the 2021 World Health Organization (WHO) classification of central nervous system tumors (13).

### 2.2 Image acquisition and assessment

All patients underwent preoperative MRI within two weeks of surgery or biopsy (**Supplementary Tables 1**, **2**). The preoperative MRI scanning protocol included pre- and post-contrast imaging. Pre-contrast imaging included sagittal and axial T1WI, axial T2WI, and FLAIR. After pre-contrast scanning, the contrast media (dimeglumine gadopentetate; 0.2 ml/kg; Beilu^®^, Beijing Beilu Pharmaceutical Co., LTD, Beijing, China) was injected into the antecubital vein. Post-contrast scanning was performed immediately thereafter, including the acquisition of sagittal, axial, and coronal T1WI images. Postoperative MRI was performed within 72 h of tumor resection or biopsy. The scanning protocol for the postoperative MRI and subsequent MRI during follow-up was the same as that for the preoperative MRI.

After the identification of the glioma type, the radiological features, including tumor location, peritumoral oedema, extent of tumor enhancement, and proportion of enhancing lesions, were evaluated for each patient. The tumor location was classified as deep (basal ganglia, internal capsules, thalamus, brainstem, corpus callosum, or subventricular zone), superficial (frontal lobe, parietal lobe, temporal lobe, occipital lobe, insula, or other regions that did not meet the criteria for deep location), or both (6, 14, 15). On T2WI and FLAIR images, hyperintense areas outside the tumor margin that correspond to hypointensity on T1WI are determined as peritumoral edema (16, 17). The maximum width of the peritumoral oedema was measured using Neurosoft PACS software (http://www.neusoft.com) on FLAIR images and was classified as zero, ≤10 mm, or >10 mm (18, 19). The extent of tumor enhancement was classified as none, mild, or marked, according to the Visually Accessible Rembrandt Images (VASARI) feature set (20). The proportion of enhancing lesions was defined as the number of enhanced lesions divided by the total number of lesions in patients with multiple gliomas.

The identification of multiple gliomas and subtyping were independently performed by two authors (LF and YZ, with two and three years of experience, respectively). Inconclusive cases were reviewed by a senior neuroradiologist (CX, with 20 years of experience). The radiological features were evaluated using Neurosoft PACS software.

### 2.3 Clinical and pathological assessment

Clinical data, including patient age, sex, and surgical data, were collected. The patient age was recorded on the date of the preoperative MRI examination. The type of surgery was classified as gross total resection (GTR), non-GTR, biopsy, and intact lesion(s) for multiple gliomas. Based on postoperative MRI imaging and surgical records, GTR was defined as the removal of >95% of the tumor mass (postoperative tumor volume/preoperative tumor volume <5%), and non-GTR was defined as subtotal resection and partial resection (6, 21, 22). Intact lesion(s) indicated that the lesion was not resected or biopsied.

Histopathological diagnoses and immunohistochemical statuses were collected from the neuropathology reports. The expression levels of alpha-thalassemia X-linked(ATR-X), P53, oligodendrocyte transcription factor 2, glial fibrillary acidic protein, epidermal growth factor receptor, vascular endothelial-derived growth factor, and phosphatase and tensin homologue were determined using immunohistochemistry (IHC). Complete deletion of both the short arm of chromosome 1 (1p) and the long arm of chromosome 19 (19q) (1p/19q codeletion) was identified using clinical fluorescence *in situ* hybridization assays. The statuses of isocitrate dehydrogenase (IDH), telomerase reverse transcriptase (TERT) promoter, and O (6)-methylguanine-DNA methyltransferase promoter methylation were determined using IHC or next-generation sequencing (23).

### 2.4 Survival assessment

Overall survival (OS) was calculated as the time between the date of neurosurgery and the date of death due to glioma. Progression-free survival (PFS) was assessed as either the time between the date of neurosurgery and tumor progression or the time between the date of neurosurgery and the date of death due to glioma (15, 24). Tumor progression was defined using MRI findings according to the response assessment in neuro-oncology criteria (25). Follow-up was conducted using the outpatient or inpatient system or via telephone when necessary. Patients with lower-grade gliomas underwent follow-up at six months while those with high-grade gliomas underwent follow-up at three months. All patients underwent surgical treatment. Patients diagnosed with WHO grade 3 and 4 gliomas, as well as those diagnosed with high-risk WHO grade 2 gliomas (defined as patients over 40 years of age or patients without total resection), were also given radiotherapy and chemotherapy. The final follow-up was conducted in October 2023, and the median follow-up time was 30 months (95% confidence interval (CI): 26.2-33.8). Loss to follow-up and survival at the last follow-up were defined as censoring.

### 2.5 Statistical analysis

Of the 955 patients with cerebral glioma included in this study, 94 had multiple gliomas and 861 had unifocal gliomas.

Random sampling was conducted in the unifocal glioma cohort by labelling the cohort according to the time order of the pathological results and generating random numbers. A total of 188 patients with unifocal gliomas were randomly selected for the analysis. Then, the data of the 188 selected patients were compared with the data of the total cohort (861 patients) to determine if the selection was random and eliminate the possibility of selection bias.

The clinical, radiological, and pathological factors of patients with unifocal and multiple gliomas were compared. Logistic regression analyses were conducted for factors with significant differences to identify independent risk factors. The characteristics of patients with multifocal and multicentric gliomas were also compared. The Shapro-Wilk test were used to check the normality of the distribution of continuous variables. Non-parametric tests (Mann-Whitney U test) were used when the distribution of a continuous variable was non-normal. Two-sample t-tests were used to compare continuous variables with normal distribution. The chi-square test or Fisher’s exact test were used to compare categorical variables, such as sex, radiological features, surgical data, histopathological diagnosis, and immunohistochemical status.

OS and PFS were assessed using Kaplan-Meier survival curves and compared using the log-rank test. Univariate survival analyses were conducted using a Cox proportional hazards regression analysis. The multivariate Cox proportional hazards regression was unreliable to assess unifocal glioma due to the multicollinearity present within the dataset. Therefore, the multivariate survival analysis performed on the unifocal glioma dataset was based on penalized Cox models from the “scikit-survival” and “scikit-learn” packages in Python (version 3.8). Multivariate survival analysis of the multiple glioma datasets was performed using Cox proportional hazards regression in SPSS (version 26; IBM, Armonk, NY, USA). Statistical significance was set at P<0.05.

## 3 Results

**Figure 1:**
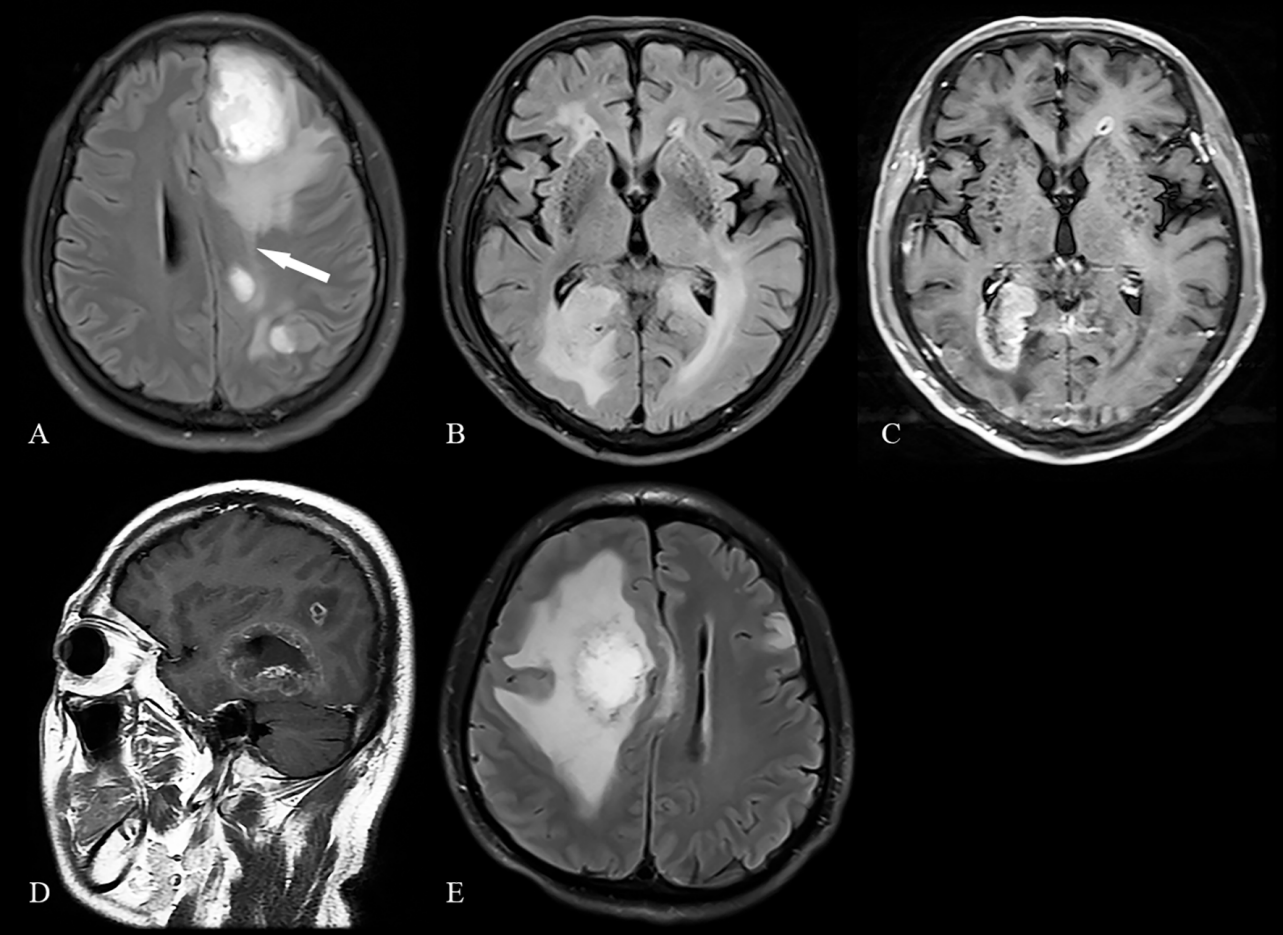
Typical manifestations of multifocal and multicentric gliomas **(A)**, Multifocal gliomas type 1: Two separate lesions communicate through white matter fibers, appearing as hyperintense signal on an axial fluid-attenuated inversion recovery (FLAIR) image (arrow). **(B, C)**, Multifocal gliomas type 2: Separate lesions abutting the ventricular system are shown on an axial FLAIR image and post-contrast T1-weighted image. **(D)**, Multifocal gliomas type 3: Satellite foci adjacent to the main tumor are shown on a sagittal post-contrast T1-weighted image. **(E)** Multicentric gliomas: Isolated lesions in different hemispheres are shown on an axial FLAIR image.

A total of 861 patients (90.2%) had unifocal gliomas and 94 (9.8%) had multiple gliomas, including 59 (62.8%) with multifocal gliomas and 35 (37.2%) with multicentric gliomas (**Figure 1**). The 188 randomly selected patients with unifocal glioma were statistically representative of the entire unifocal glioma cohort (**Supplementary Table 3**), eliminating the risk of selection bias.

### 3.1 Clinical, pathological, and radiological characteristics of patients with unifocal and multiple gliomas

Patients with multiple gliomas were older (P=0.001) and more likely to be male (χ2 = 4.857, P=0.028) than those with unifocal gliomas. Patients with multiple gliomas underwent smaller extent of surgical resection (χ2 = 161.016, P<0.001) and had a worse prognosis (χ2 = 43.733, P< 0.001) than those with unifocal gliomas.

A non-superficial location (χ2 = 51.758, P<0.001), severe peritumoral oedema (χ2 = 9.688, P=0.008), and increased enhancement intensity (χ2 = 24.547, P<0.001) were more common in the multiple gliomas group.

Patients in the multiple gliomas group had higher WHO grades (P=0.001) and a lower ratio of IDH mutation (χ2 = 51.770, P<0.001) and rate of 1p19q codeletion (χ2 = 8.637, P=0.003) than those in the unifocal gliomas group (**Table 1**).

**Table 1:** Clinical, radiological and pathological characteristics of studied groups.

Characteristics	Unifocal (n=188)	Multiple (n=94)	*p* Value	Chi-square value	Multifocal (n=59)	Multicentric (n=35)	*p* Value	Chi-square value
Age (y), mean ± SD	46.5 ± 13.6	52.4 ± 14.3	**0.001^b^**		54.1 ± 12.6	49.6 ± 16.7	0.178^b^	
Sex			**0.028**	4.857			**0.011**	6.521
Male	96 (51.1%)	61 (64.9%)			44 (74.6%)	17 (48.6%)		
Female	92 (48.9%)	33 (35.1%)			15 (25.4%)	18 (51.4%)		
Surgical treatment			**<0.001**	161.016			0.451^a^	
Biopsy	11 (5.9%)	22 (23.4%)			13 (22%)	9 (25.7%)		
non-GTR	64 (34.0%)	7 (7.4%)			5 (8.5%)	2 (5.7%)		
GTR	113 (60.1%)	15 (16.0%)			12 (20.3%)	3 (8.6%)		
Lesion (s) left	–	50 (53.2%)			29 (49.2%)	21 (60.0%)		
Survival status			**<0.001**	43.733			0.753	0.568
Died	38 (20.2%)	49 (52.1%)			30 (50.8%)	19 (54.3%)		
Alive	99 (52.7%)	14 (14.9%)			8 (13.6%)	6 (17.1%)		
NA	51 (27.1%)	31 (33.0%)			21 (35.6%)	10 (28.6%)		
Location			**<0.001**	51.758			**0.048^a^**	
Superficial	146 (77.7%)	36 (38.3%)			17 (28.8%)	19 (54.3%)		
Deep	12 (6.4%)	4 (4.3%)			3 (5.1%)	1 (2.9%)		
Both	30 (16.0%)	54 (57.4%)			39 (66.1%)	15 (42.9%)		
Edema			**0.008**	9.688			0.05^a^	
0mm	132 (70.2%)	49 (52.1%)			25 (42.4%)	24 (68.6%)		
10mm≤	8 (4.3%)	4 (4.3%)			3 (5.1%)	1 (2.9%)		
>10mm	48 (25.5%)	41 (43.6%)			31 (52.5%)	10 (28.6%)		
Enhancement intensity			**<0.001**	24.547			0.119	4.254
None	71 (37.8%)	15 (16.0%)			11 (18.6%)	4 (11.4%)		
Mild	38 (20.2%)	11 (11.7%)			4 (6.8%)	7 (20.0%)		
Marked	75 (39.9%)	67 (71.3%)			44 (74.6%)	23 (65.7%)		
NA	4 (2.1%)	1 (1.1%)			0	1 (2.9%)		
Proportion of enhancing lesion (%)			–				0.379^c^	
Median	–	50			66.7	50.0		
WHO grades			**0.001^a^**				0.122	4.204
Grade1	2 (1.1%)	0			0	0		
Grade2	66 (35.1%)	17 (18.1%)			9 (15.3%)	8 (22.9%)		
Grade3	39 (20.7%)	14 (14.9%)			6 (10.2%)	8 (22.9%)		
Grade4	78 (41.5%)	62 (66.0%)			43 (72.9%)	19 (54.3%)		
NOS	3 (1.6%)	1 (1.1%)			1 (1.7%)			
IDH1/2			**<0.001**	51.770			0.738^a^	
Mutation	103 (55.7%)	10 (10.8%)			7 (12.1%)	3 (8.6%)		
Wild type	82 (44.3%)	83 (89.2%)			51 (87.9%)	32 (91.4%)		
1p19q			**0.003**	8.637			0.290^a^	
Noncodeletion	100 (72.5%)	48 (92.3%)			31 (88.6%)	17 (100%)		
Codeletion	38 (27.5%)	4 (7.7%)			4 (11.4%)	0		
TERT promoter			0.269	1.220			0.245^a^	
Mutation	75 (55.1%)	24 (46.2%)			18 (52.9%)	6 (33.3%)		
Wild type	61 (44.9%)	28 (53.8%)			16 (47.1%)	12 (66.7%)		
MGMT promoter			0.810	0.058			0.571^a^	
Methylated	100 (69.9%)	43 (68.3%)			31 (70.5%)	12 (63.2%)		
Unmethylated	43 (30.1%)	20 (31.7%)			13 (29.5%)	7 (36.8%)		
ATRX			0.502	0.451			0.729^a^	
Intact	122 (80.8%)	60 (84.5%)			42 (85.7%)	18 (81.8%)		
Loss	29 (19.2%)	11 (15.5%)			7 (14.3%)	4 (18.2%)		
P53			0.240	1.382			0.737^a^	
Positive	125 (79.1%)	65 (85.5%)			41 (83.7%)	24 (88.9%)		
Negative	33 (20.9%)	11 (14.5%)			8 (16.3%)	3 (11.1%)		
Olig2			0.380^a^				1^a^	
Positive	111 (100%)	67 (98.5%)			44 (97.8%)	23 (100%)		
Negative	0	1 (1.5%)			1 (2.2%)	0		
GFAP			1^a^				–	
Positive	105 (99.1%)	65 (100%)			45 (100%)	20 (100%)		
Negative	1 (0.9%)	0			0	0		

ATRX, alpha-thalassemia X-linked; GFAP, glial fibrillary acidic protein; GTR, gross total resection; IDH, isocitrate dehydrogenase; MGMT, O6-methylguanine-DNA methyltransferase; NA, not available data; Olig2, oligodendrocyte transcription factor 2; OS, overall survival; PFS, progression free time; SD, standard deviation; TERT, telomerase reverse transcriptase.

^a^p Value from Fisher exact test.

^b^P Value from 2-sample t test.

^c^p Value from Mann-Whitney U test.

Bold p Value < 0.05 is considered statistically significant

After multivariate logistic regression analysis, tumor location and IDH status were identified as independent risk factors for multiple gliomas (P <0.001 and P =0.003, respectively) (**Supplementary Table 4**). The model demonstrated an accuracy (ACC) of 0.82 (95% CI: 0.73–0.92), a sensitivity (SEN) of 0.38 (95% CI: 0.12–0.65), and a specificity (SPE) of 0.95 (95% CI: 0.89–1.00).

### 3.2 Clinical, pathological, and radiological characteristics of patients with multifocal and multicentric gliomas

Patients with multifocal gliomas were more likely to be male than those with multicentric gliomas (χ2 = 6.521, P=0.011). Multifocal gliomas tended to involve both the superficial and deep cerebral parenchyma simultaneously (66.1%, 39/59), while multicentric gliomas were more likely to be located superficially (54.3%, 19/35) (P=0.048). There were no differences in genetic expressions between the two types of gliomas (**Table 1**).

### 3.3 Survival analysis of patients with unifocal and multiple gliomas

**Figure 2:**
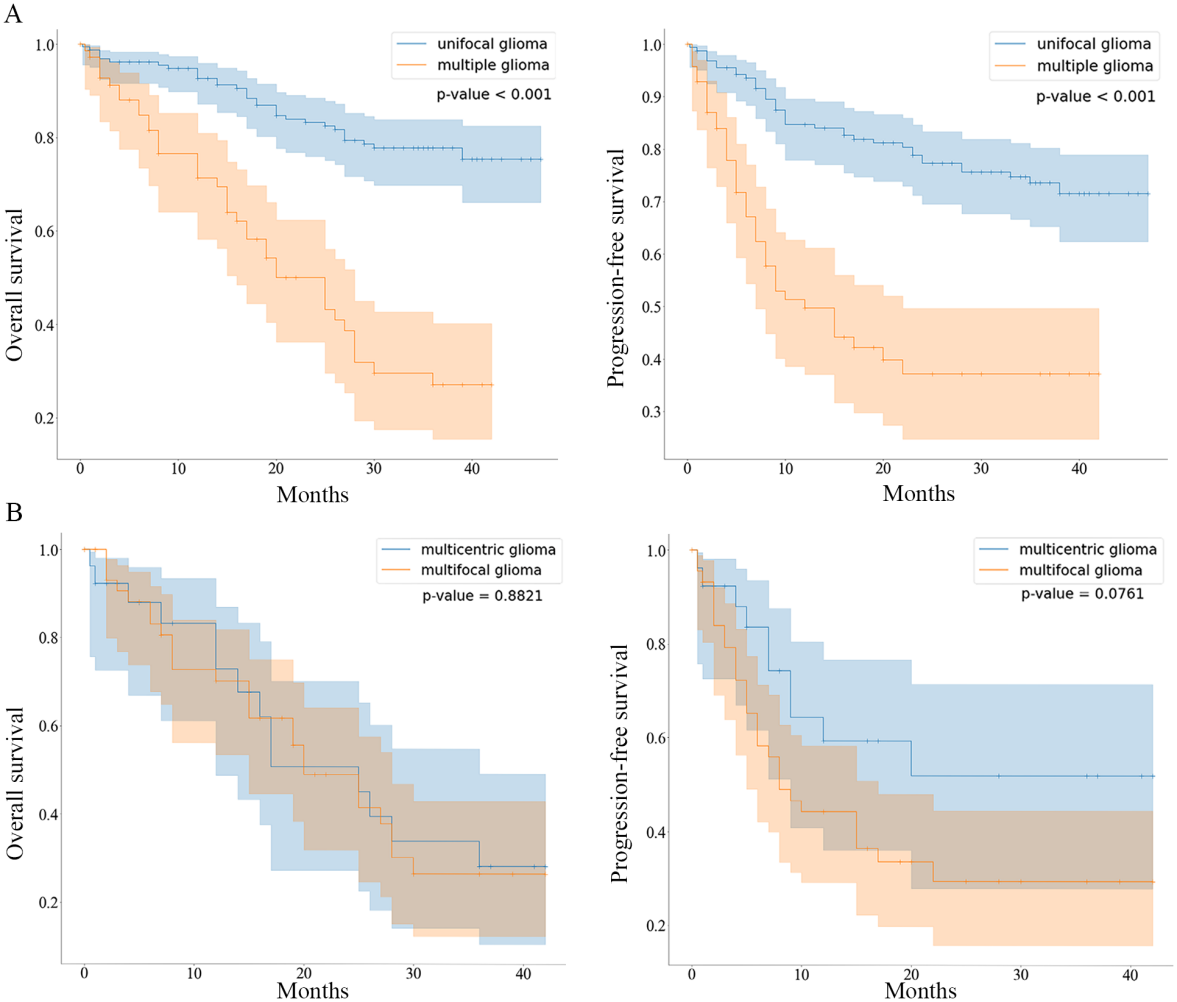
Kaplan-Meier curves comparing OS and PFS between patients with unifocal/multiple gliomas and patients with multifocal/multicentric gliomas **(A)**, Kaplan-Meier curves comparing the overall survival (OS) and progression-free survival (PFS) of the unifocal and multiple gliomas groups are shown. **(B)**, Kaplan-Meier curves comparing the OS and PFS of the multifocal and multicentric gliomas groups are shown.

At the end of the follow-up period, 52.7% (99/188) of the patients with unifocal gliomas and 14.9% (14/94) of the patients with multiple gliomas were alive. The median OS and PFS of patients with unifocal gliomas were not determined as over half of the patients had not experienced the respective outcome events at the end of the study. The median OS of patients with multiple gliomas was 25 months (95% CI: 17.81–32.19 months) and the median PFS was 12 months (95% CI: 5.70–18.30 months). The three-year OS rates in the unifocal and multiple gliomas groups were 73.6% and 37.2%, respectively. Patients with unifocal gliomas had significantly longer OS and PFS (P<0.001 and P<0.001, respectively) than those with multiple gliomas (**Figure 2A**).

**Figure 3:**
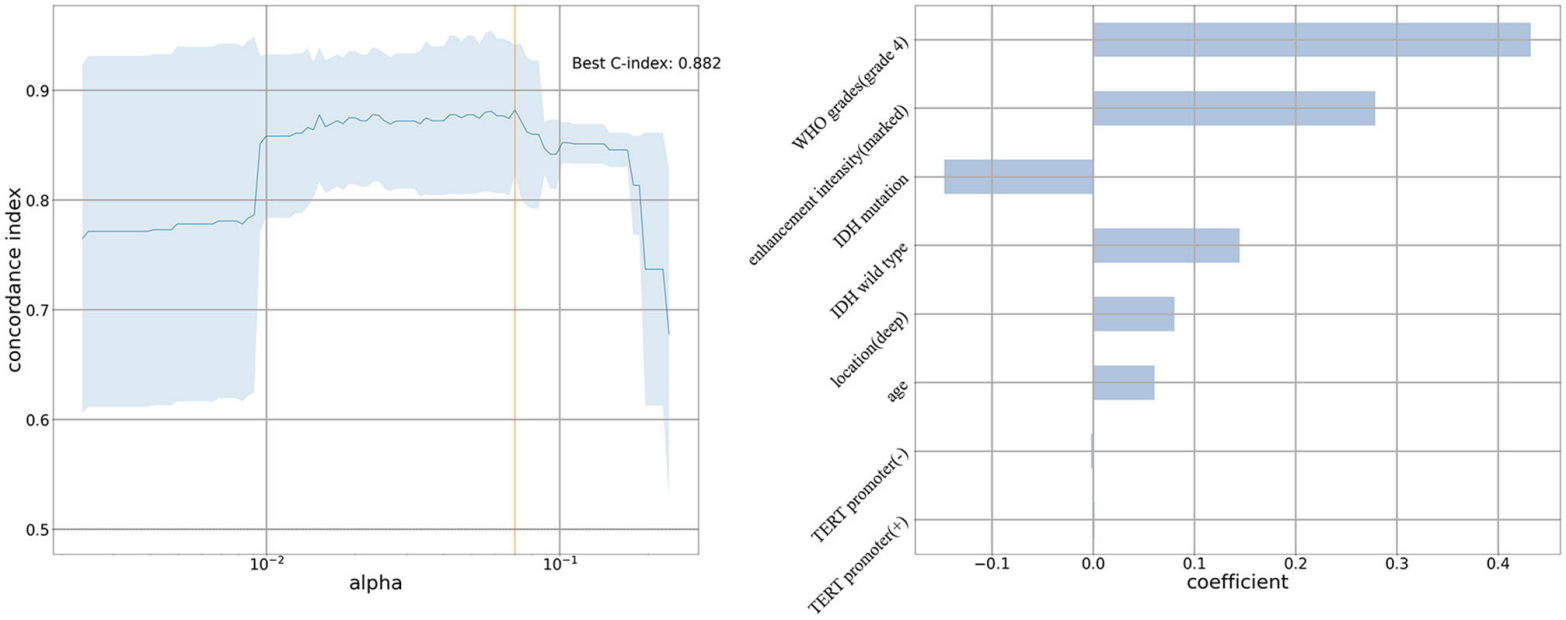
Penalized cox model of unifocal glioma group The C-index is 0.882. Patient age, deep tumor location, World Health Organization grade 4, marked enhancement intensity, IDH (mutation/wild type), and TERT promoter mutation status (positive/negative) are independent risk factors for overall survival in the unifocal glioma group.

Univariable Cox regression analysis showed that patients with unifocal and multiple gliomas had five similar risk factors (age, tumor location, peritumoral edema, enhancement intensity, WHO grades) and four different risk factors (extent of resection, 1p19q/IDH/TERT status) (**Supplementary Tables 5**, **6**). After selecting factors in penalized cox models, patient age, deep tumor location, WHO grade 4, marked enhancement intensity, IDH (mutation/wild type), and TERT promoter mutation status (positive/negative) were identified as independent risk factors for OS in the unifocal glioma group (**Figure 3**). And multivariable Cox regression analysis found that deep tumor location was associated with a shorter OS in the multiple gliomas group (hazard ratio: 3.365, 95% CI: 1.061–10.676, P=0.039) (**Table 2**).

**Table 2:** Multivariate cox regression analysis of OS in multiple gliomas patients.

Characteristics	Multivariable analysis	
	Hazard ratio (95%CI)	*p* Value
Age	1.027 (0.994-1.061)	0.116
Location		
Superficial	1	0.083
Deep	3.365 (1.061, 10.676)	**0.039**
Both	0.626 (0.144,2.709)	0.530
Edema		
0mm	1	0.423
10mm≤	2.337 (0.513,10.648)	0.273
>10mm	0.970 (0.394,2.391)	0.948
Enhancement intensity		
None	1	0.450
Mild	0.658 (0.058,7.517)	0.736
Marked	3.150 (0.312,31.835)	0.331
WHO grades		
Grade1	<0.001	0.982
Grade2	0.257 (0.012,5.364)	0.381
Grade3	0.659 (0.079,5.527)	0.701
Grade4	1	0.852
IDH1/2 (reference to wild type)	2.999 (0.571,15.739)	0.194

The multivariate Cox regression analysis is used for variables with p Value < 0.1 in univariate analysis. Bold p Value < 0.05 is considered statistically significant.

### 3.4 Survival analysis of patients with multifocal and multicentric gliomas

The mortality rates at the last follow-up were 50.8% (30/59) and 54.3% (19/35) in the multifocal and multicentric gliomas groups, respectively (P=0.753). The OS and PFS rates were not significantly different between the two groups (P=0.882 and P=0.076, respectively) (**Figure 2B**).

No variable was identified as risk factor in the multifocal gliomas group (**Supplementary Figure 2A**). The WHO grade was identified as a prognostic factor in the multicentric gliomas group (P=0.034) (**Supplementary Figure 2B**).

### 3.5 Analysis of 15 patients underwent surgical treatment for more than one lesion

Of all the 94 multiple patients, 15 patients (8, multifocal gliomas; 7, multicentric gliomas) underwent surgical resection or biopsy for more than one lesion (**Supplementary Table 7**). And the pathological results showed that, in one patient, the different lesions can be the same or different WHO grades. Four of the 7 patients were still alive at the end of the follow-up. They were younger (mean age: 35.75 vs. 54.91 years) and had a larger proportion of lower grade glioma (75.0% vs. 0%) than the rest 11 patients.

## 4 Discussion

The current study identified differences in the clinical, radiological, and pathological characteristics of patients with multiple and unifocal gliomas. Patients with multiple gliomas are older, predominantly male, more likely to undergo a smaller extent of surgical resection and have a poor prognosis than those with unifocal gliomas. Most of these results were consistent with those of previous studies (9, 26, 27).

The location of lesions were different between the multiple and unifocal gliomas groups, and patients with multiple gliomas were more likely to have obvious peritumoral oedema and more intense enhancement on MRI. The location of the lesions in patients with multiple gliomas in the current study differed from that in a previous study (6), which may be due to different types of gliomas. In the current study, WHO grades 2–4 gliomas were included, though only glioblastomas (WHO grade 4) were included in the previous study (6). The tendencies of patients with multiple gliomas to have obvious peritumoral oedema and increased enhancement intensity have not been reported previously. However, these findings are not surprising as multiple gliomas have a high ratio of high-grade gliomas (80.9% vs. 62.2% of unifocal gliomas) and these two radiological signs indicate that the tumor has malignant pathological behavior (17, 24).

In the current study, patients with multiple gliomas had a lower ratio of IDH mutation (χ2 = 51.770, P<0.001) and 1p19q codeletion (χ2 = 8.637, P=0.003). The 1p19q status has been compared between glioma types in a previous study (28), though this is the first report comparing the IDH status between these groups. However, the pathogenesis of multiple gliomas remains poorly understood. The findings of the current study combined with those of previous studies (29–31) indicate that multiple gliomas have a different pathogenesis than unifocal gliomas. In addition, the tumor location and IDH status were identified as independent risk factors for multiple gliomas in our study, which may improve the understanding of the mechanisms underlying the occurrence of multiple gliomas.

In the current study, the sex and tumor location differed between patients with multifocal and multicentric gliomas. Patients with multifocal gliomas were predominantly male, and patients with multicentric gliomas were more likely to have only superficial lesions(54.3% vs. 28.8%). These demographic and anatomical differences between the two subtypes of multiple gliomas indicate that there may be different mechanisms of occurrence between these two types of tumors. Additionally, we note that the conventional imaging features of multicentric and multifocal gliomas show no significant differences, and whether histological connections exist in some multicentric gliomas that cannot be detected by conventional imaging methods still requires further exploration with advanced imaging techniques.

Patients with multiple gliomas had a poorer prognosis than those with unifocal gliomas, though the prognosis was not significantly different between patients with multifocal and multicentric gliomas in the current study, which is in accordance with the results of previous studies (5, 8). The prognostic risk factors were further explored in the current study, and only deep tumor location was identified as an independent risk factor for shorter OS in patients with multiple gliomas. This finding indicates that lesions in deep anatomical areas must be treated differently than those in superficial locations. Furthermore, after analyzing the two subtypes of multiple gliomas, we found that no variables were identified as prognostic risk factors for multifocal gliomas. However, the WHO grades were found to be a significant prognostic factor for multicentric gliomas. This suggests that clinicians should pay attention to distinguishing between these two subtypes in clinical practice.

This study is not without limitations. First, as this was a retrospective study, bias may have been present. Second, radiological analysis was based on routine MRI. Advanced MRI, such as diffusion weighted imaging and magnetic resonance spectroscopy, was not available. These advanced MRI modalities may provide more information regarding gliomas and should be considered in future studies. Third, molecular indices such as the Ki-67 levels were not analyzed. However, some genes were analyzed in the current study. Therefore, molecular indices should also be considered in future studies.

In conclusion, the clinical, radiological, and pathological characteristics of patients with unifocal and multiple gliomas and patients with multifocal and multicentric gliomas were compared in the current study. Patients with multiple gliomas differ from those with unifocal gliomas in terms of demographic characteristics, surgery, and prognosis. Clinical, radiological, and pathological features differ between patients with unifocal and multiple gliomas and patients with multifocal and multicentric gliomas.

## Data Availability

The raw data supporting the conclusions of this article will be made available by the authors, without undue reservation.

## Supplementary material

Supplementary Figure 1 Flow chart of patient selection process.

Supplementary Figure 2 Kaplan-Meier curves comparing OS for different characteristics in multifocal and multicentric gliomas groups **(A)**, Multifocal gliomas group: No overall survival risk factors are identified. **(B)**, Multicentric gliomas group: The World Health Organization grade is significantly associated with overall survival.

## References

[1] Davis ME. Epidemiology and overview of gliomas. Semin Oncol Nurs. (2018) 34:420–9. doi: 10.1016/j.soncn.2018.10.001 30392758

[2] Mo Z Xin J Chai R Woo PYM Chan DTM Wang J. Epidemiological characteristics and genetic alterations in adult diffuse glioma in East Asian populations. Cancer Biol Med. (2022) 19:1440–59. doi: 10.20892/j.issn.2095-3941.2022.0418 PMC9630523 36350002

[3] Nicholson JG Fine HA. Diffuse glioma heterogeneity and its therapeutic implications. Cancer Discov. (2021) 11:575–90. doi: 10.1158/2159-8290.CD-20-1474 33558264

[4] Barthel L Hadamitzky M Dammann P Schedlowski M Sure U Thakur BK. Glioma: molecular signature and crossroads with tumor microenvironment. Cancer Metastasis Rev. (2022) 41:53–75. doi: 10.1007/s10555-021-09997-9 34687436 PMC8924130

[5] Xiong YJ Zhao XL Wang XY Pan DJ Tian DS. Multiple cerebral gliomas mimicking central nervous system inflammatory demyelinating diseases: A rare case with review of literature. Med (Baltimore). (2017) 96:e9456. doi: 10.1097/MD.0000000000009456 PMC6392929 29384930

[6] Lasocki A Gaillard F Tacey M Drummond K Stuckey S. Multifocal and multicentric glioblastoma: Improved characterisation with FLAIR imaging and prognostic implications. J Clin Neurosci. (2016) 31:92–8. doi: 10.1016/j.jocn.2016.02.022 27343042

[7] Aure K Laigle-Donadey F Kaloshi G Amiel-Benouaich A Sanson M. Multiple gliomas: clinical studies and pathophysiological hypothesis. Rev Neurol (Paris). (2006) 162:845–51. doi: 10.1016/s0035-3787(06)75088-3 17028546

[8] Di Carlo DT Cagnazzo F Benedetto N Morganti R Perrini P. Multiple high-grade gliomas: epidemiology, management, and outcome. A systematic review and meta-analysis. Neurosurg Rev. (2019) 42:263–75. doi: 10.1007/s10143-017-0928-7 29138949

[9] Huang R Wu H Lu X Sun X. Clinical characteristics and prognostic factors of solitary and multiple adult gliomas: a retrospective study based on propensity score matching. Eur Rev Med Pharmacol Sci. (2023) 27:10481–98. doi: 10.26355/eurrev_202311_34325 37975372

[10] Huang YH Feng QJ. Segmentation of brain tumor on magnetic resonance images using 3D full-convolutional densely connected convolutional networks. Nan Fang Yi Ke Da Xue Xue Bao. (2018) 38:661–8. doi: 10.3969/j.issn.1673-4254.2018.06.04 PMC6765705 29997087

[11] Kasper J Hilbert N Wende T Fehrenbach MK Wilhelmy F Jahne K. On the prognosis of multifocal glioblastoma: an evaluation incorporating volumetric MRI. Curr Oncol. (2021) 28:1437–46. doi: 10.3390/curroncol28020136 PMC8167648 33917207

[12] Perez-Beteta J Molina-Garcia D Villena M Rodriguez MJ Velasquez C Martino J. Morphologic features on MR imaging classify multifocal glioblastomas in different prognostic groups. AJNR Am J Neuroradiol. (2019) 40:634–40. doi: 10.3174/ajnr.A6019 PMC7048517 30923085

[13] Louis DN Perry A Wesseling P Brat DJ Cree IA Figarella-Branger D. The 2021 WHO classification of tumors of the central nervous system: a summary. Neuro Oncol. (2021) 23:1231–51. doi: 10.1093/neuonc/noab106 PMC8328013 34185076

[14] Bao H Wang H Sun Q Wang Y Liu H Liang P. The involvement of brain regions associated with lower KPS and shorter survival time predicts a poor prognosis in glioma. Front Neurol. (2023) 14:1264322. doi: 10.3389/fneur.2023.1264322 38111796 PMC10725945

[15] Fadel HA Haider S Pawloski JA Zakaria HM Macki M Bartlett S. Laser interstitial thermal therapy for first-line treatment of surgically accessible recurrent glioblastoma: outcomes compared with a surgical cohort. Neurosurgery. (2022) 91:701–9. doi: 10.1227/neu.0000000000002093 35986677

[16] Ohmura K Tomita H Hara A. Peritumoral edema in gliomas: A review of mechanisms and management. Biomedicines. (2023) 11(10):2731. doi: 10.3390/biomedicines11102731 37893105 PMC10604286

[17] Han Q Lu Y Wang D Li X Ruan Z Mei N. Glioblastomas with and without peritumoral fluid-attenuated inversion recovery (FLAIR) hyperintensity present morphological and microstructural differences on conventional MR images. Eur Radiol. (2023) 33:9139–51. doi: 10.1007/s00330-023-09924-2 37495706

[18] Lasocki A Gaillard F. Non-contrast-enhancing tumor: A new frontier in glioblastoma research. AJNR Am J Neuroradiol. (2019) 40:758–65. doi: 10.3174/ajnr.A6025 PMC7053910 30948373

[19] Uetani H Azuma M Khant ZA Watanabe Y Kudo K Kadota Y. Importance of age and noncontrast-enhancing tumor as biomarkers for isocitrate dehydrogenase-mutant glioblastoma: A multicenter study. J Comput Assist Tomogr. (2023) 47:659–65. doi: 10.1097/RCT.0000000000001456 PMC10348614 36877775

[20] Cancer Imaging Archive. VASARI research project (2019). Available at: https://wiki.cancerimagingarchive.net/display/Public/VASARI+Research+Project (Accessed February 20, 2023).

[21] Lasocki A Gaillard F Tacey MA Drummond KJ Stuckey SL. The incidence and significance of multicentric noncontrast-enhancing lesions distant from a histologically-proven glioblastoma. J Neurooncol. (2016) 129:471–8. doi: 10.1007/s11060-016-2193-y 27412000

[22] Terakawa Y Yordanova YN Tate MC Duffau H. Surgical management of multicentric diffuse low-grade gliomas: functional and oncological outcomes: clinical article. J Neurosurg. (2013) 118:1169–75. doi: 10.3171/2013.2.JNS121747 23495876

[23] Wu M Jiang T Guo M Duan Y Zhuo Z Weng J. Amide proton transfer-weighted imaging and derived radiomics in the classification of adult-type diffuse gliomas. Eur Radiol. (2024) 34:2986–96. doi: 10.1007/s00330-023-10343-6 37855851

[24] Patel SH Batchala PP Muttikkal TJE Ferrante SS Patrie JT Fadul CE. Fluid attenuation in non-contrast-enhancing tumor (nCET): an MRI Marker for Isocitrate Dehydrogenase (IDH) mutation in Glioblastoma. J Neurooncol. (2021) 152:523–31. doi: 10.1007/s11060-021-03720-y 33661425

[25] Wen PY van den Bent M Youssef G Cloughesy TF Ellingson BM Weller M. RANO 2.0: Update to the Response Assessment in Neuro-Oncology Criteria for High- and Low-Grade Gliomas in Adults. J Clin Oncol. (2023) 41:5187–99. doi: 10.1200/JCO.23.01059 PMC10860967 37774317

[26] Wang R Song Y Hu T Wang X Jiang Y Zhang D. Decreased CD8(+) lymphocytic infiltration in multifocal and multicentric glioblastomas. Front Oncol. (2021) 11:748277. doi: 10.3389/fonc.2021.748277 34646781 PMC8503598

[27] Haque W Thong Y Verma V Rostomily R Brian Butler E Teh BS. Patterns of management and outcomes of unifocal versus multifocal glioblastoma. J Clin Neurosci. (2020) 74:155–9. doi: 10.1016/j.jocn.2020.01.086 32089384

[28] Karlowee V Amatya VJ Hirano H Takayasu T Nosaka R Kolakshyapati M. Multicentric glioma develops via a mutant IDH1-independent pathway: immunohistochemical study of multicentric glioma. Pathobiology. (2017) 84:99–107. doi: 10.1159/000447951 27553586

[29] Dono A Wang E Lopez-Rivera V Ramesh AV Tandon N Ballester LY. Molecular characteristics and clinical features of multifocal glioblastoma. J Neurooncol. (2020) 148:389–97. doi: 10.1007/s11060-020-03539-z 32440969

[30] Abou-El-Ardat K Seifert M Becker K Eisenreich S Lehmann M Hackmann K. Comprehensive molecular characterization of multifocal glioblastoma proves its monoclonal origin and reveals novel insights into clonal evolution and heterogeneity of glioblastomas. Neuro-Oncology. (2017) 19:546–57. doi: 10.1093/neuonc/now231 PMC5464316 28201779

[31] Wu L Wu W Zhang J Zhao Z Li L Zhu M. Natural coevolution of tumor and immunoenvironment in glioblastoma. Cancer Discov. (2022) 12:2820–37. doi: 10.1158/2159-8290.CD-22-0196 PMC9716251 36122307

