# Supplementary figures and images for: Different risk factors for multiple and unifocal gliomas: a comparative study of radiological, pathological and clinical characteristics
Source: Front Oncol. 2025 May 27;15:1531879. doi: 10.3389/fonc.2025.1531879 (PMC12149172; doi:10.3389/fonc.2025.1531879)

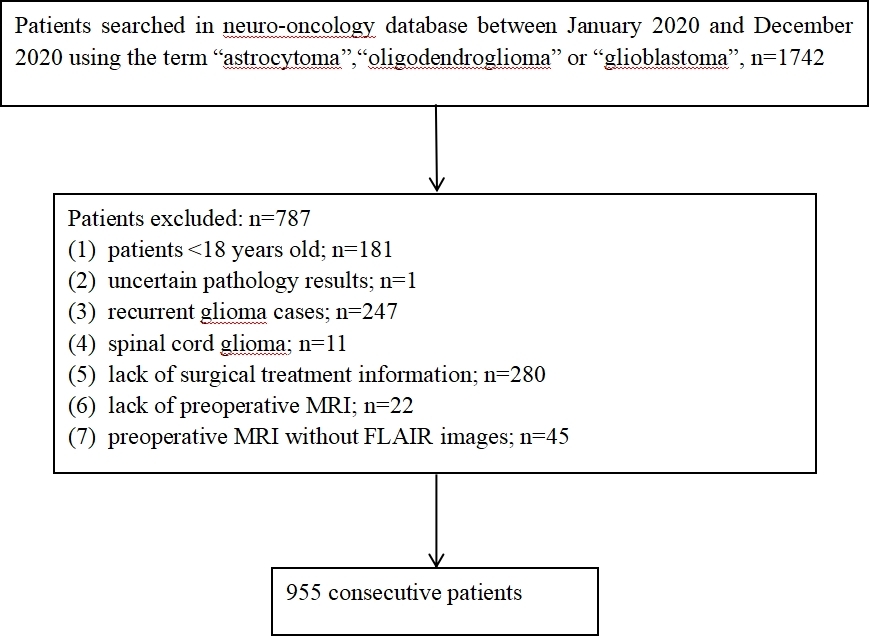

Supplement: Supplementary Figure 1 — Flow chart of patient selection process. [file Image1.jpeg]

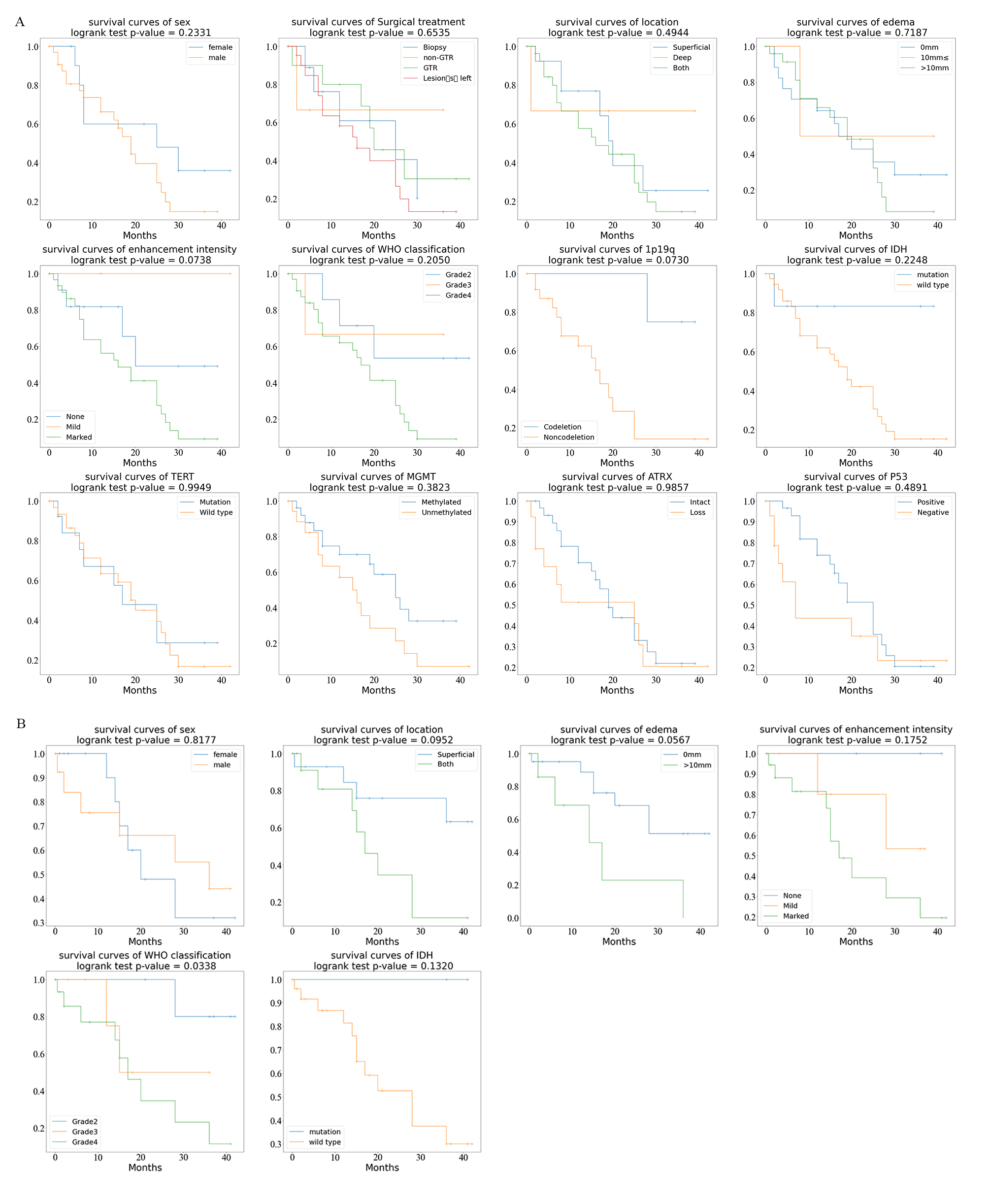

Supplement: Supplementary Figure 2 — Kaplan-Meier curves comparing OS for different characteristics in multifocal and multicentric gliomas groups (A), Multifocal gliomas group: No overall survival risk factors are identified. (B), Multicentric gliomas group: The World Health Organization grade is significantly associated with overall survival. [file Image2.tif]
