# Supplementary material for: Different risk factors for multiple and unifocal gliomas: a comparative study of radiological, pathological and clinical characteristics
Source: Front Oncol. 2025 May 27;15:1531879. doi: 10.3389/fonc.2025.1531879 (PMC12149172; doi:10.3389/fonc.2025.1531879)
Supplement: Supplementary file 3 [file Table1.docx]

**Supplementary Table 1** The MR scanners used in preoperative scanning

| MR scanners | Cases(n) |
| --- | --- |
| GE Discovery MR750 | 33 |
| GE SIGNA Explorer | 96 |
| Siemens MAGNETOM Prisma | 118 |
| Siemens MAGNETOM Verio | 288 |
| Philips Ingenia CX | 178 |
| Philips Ingenia | 242 |
| Summary | 955 |

**Supplementary Table 2** The MRI scanning parameters

| MR scanners | T1WI | | | |
| --- | --- | --- | --- | --- |
|  | TR(ms) | TE(ms) | Slice/Gap (mm) | Matrix |
| GE Discovery MR750 | 2275 | 19-29 | 5.50/6.50 | 512×512 |
| GE SIGNA Explorer | 2113-2115 | 29-30 | 5.50/6.50 | 512×512 |
| Siemens MAGNETOM Prisma | 1560 | 1-2 | 5.00/6.50 | 230×230 |
| Siemens MAGNETOM Verio | 1900 | 8.6 | 5.00/6.50 | 512×496 |
| Philips Ingenia CX | 6-7 | 3-4 | 5.00/6.00 | 512×512 |
| Philips Ingenia | 6-7 | 3-4 | 5.00/6.00 | 512×512 |
|  | T2WI | | | |
| GE Discovery MR750 | 6711-7385 | 106-111 | 5.50/6.50 | 512×512 |
| GE SIGNA Explorer | 4468-4473 | 90-92 | 5.50/6.50 | 512×512 |
| Siemens MAGNETOM Prisma | 5020 | 105 | 5.00/6.50 | 448×448 |
| Siemens MAGNETOM Verio | 4500 | 99 | 5.00/6.50 | 640×640 |
| Philips Ingenia CX | 4600 | 106-108 | 5.00/6.00 | 512×512 |
| Philips Ingenia | 3100 | 114-115 | 5.00/6.00 | 512×512 |
|  | FLAIR | | | |
| GE Discovery MR750 | 8000 | 147-150 | 5.50/6.50 | 512×512 |
| GE SIGNA Explorer | 8780-8811 | 140-142 | 5.50/6.50 | 512×512 |
| Siemens MAGNETOM Prisma | 5000-6800 | 81-581 | 5.00/6.50 | 320×320 |
| Siemens MAGNETOM Verio | 7500 | 85 | 5.00/6.50 | 512×464 |
| Philips Ingenia CX | 4800 | 256-257 | 5.00/6.00 | 512×512 |
| Philips Ingenia | 4800 | 278-279 | 5.00/6.00 | 512×512 |
|  | Contrast-enhanced axial T1WI | | | |
| GE Discovery MR750 | 1777-1780 | 19-20 | 5.50/6.50 | 512×512 |
| GE SIGNA Explorer | 1771-1774 | 29-30 | 5.50/6.50 | 512×512 |
| Siemens MAGNETOM Prisma | 2190 | 9 | 5.00/6.00 | 320×320 |
| Siemens MAGNETOM Verio | 2120 | 8.6 | 5.00/6.50 | 512×496 |
| Philips Ingenia CX | 2000 | 20 | 5.00/6.00 | 512×512 |
| Philips Ingenia | 2000 | 20 | 5.00/6.00 | 512×512 |

**Supplementary table 3** Comparison of randomly selected unifocal glioma samples and the cohort of the unifocal glioma

| Characteristics | Sample(n=188) | Cohort (n=861) | *p* Value | Chi-square value |
| --- | --- | --- | --- | --- |
| Age (y) |  |  | 0.523 |  |
| Range | 19-79 | 18-85 |  |  |
| Mean±SD | 46.5±13.6 | 45.8±13.3 |  |  |
| Sex |  |  | 0.128 | 2.315 |
| Male | 96(51.1%) | 492(57.1%) |  |  |
| Female | 92(48.9%) | 369(42.9%) |  |  |
| WHO grades |  |  | 0.436 | 2.725 |
| Grade1 | 2(1.1%) | 9(1.0%) |  |  |
| Grade2 | 66(35.1%) | 345(40.1%) |  |  |
| Grade3 | 39(20.7%) | 193(22.4%) |  |  |
| Grade4 | 78(41.5%) | 305(35.4%) |  |  |
| NOS | 3(1.6%) | 9(1.0%) |  |  |

**Supplementary table 4** Results of the multivariate logistic regression analysis of clinical, radiological, and pathological factors associated with multiple gliomas

| Characteristics | B value | Odds Ratio (95%CI) | *p* Value |
| --- | --- | --- | --- |
| Age | 0.007 | 1.007(0.977,1.039) | 0.647 |
| Sex (male vs. female) | -0.802 | 0.448(0.197,1.020) | 0.056 |
| Location |  |  |  |
| Superficial |  |  | **<0.001** |
| Deep | -0.329 | 0.720(0.072,7.196) | 0.780 |
| Both | 1.828 | 6.223(2.638,14.677) | **<0.001** |
| Edema |  |  |  |
| 0mm |  |  | 0.823 |
| 10mm≤ | -0.540 | 0.582(0.087,3.898) | 0.577 |
| >10mm | 0.071 | 1.074(0.376,3.067) | 0.894 |
| Enhancement intensity |  |  |  |
| None |  |  | 0.649 |
| Mild | 0.459 | 1.582(0.394,6.343) | 0.518 |
| Marked | 0.661 | 1.937(0.440,8.532) | 0.382 |
| WHO grades |  |  |  |
| Grade2 |  |  | 0.169 |
| Grade3 | -1.223 | 0.294(0.069,1.249) | 0.097 |
| Grade4 | -1.348 | 0.260(0.050,1.347) | 0.108 |
| IDH1/2 (mutation vs. wild type) | 1.835 | 6.266(1.860,21.106) | **0.003** |
| 1p19q (noncodeletion vs. codeleltion) | 1.070 | 2.915(0.657,12.934) | 0.159 |

Bold *p* Value < 0.05 is considered statistically significant

**Supplementary table 5** Univariate Cox regression analysis of OS in the unifocal glioma cohort

| Variables | HR(95%CI) | *p* Value |
| --- | --- | --- |
| Age | 1.068(1.036,1.100) | **<0.001** |
| Sex | 0.969(0.458,1.939) | 0.930 |
| Surgical treatment |  |  |
| Biopsy | 1 | 0.067 |
| non-GTR | 0.181(0.039,0.833) | **0.028** |
| GTR | 0.181(0.042,0.781) | **0.022** |
| Lesion (s) left |  |  |
| Location |  |  |
| Superficial | 1 | **<0.001** |
| Deep | 7.375(2.788,19.506) | **<0.001** |
| Both | 0.529(0.125,2.233) | 0.386 |
| Edema |  |  |
| 0mm | 1 | **<0.001** |
| 10mm≤ | 8.847(2.461,31.804) | **0.001** |
| >10mm | 4.881(2.339,10.188) | **<0.001** |
| Enhancement intensity |  |  |
| None | 1 | **<0.001** |
| Mild | 0.956(0.087,10.545) | 0.971 |
| Marked | 23.941(5.697,100.608) | **<0.001** |
| WHO grades |  |  |
| Grade1 | <0.001 | 0.981 |
| Grade2 | 0.024(0.003,0.176) | **<0.001** |
| Grade3 | 0.088(0.021,0.372) | **0.001** |
| Grade4 | 1 | **<0.001** |
| 1p19q (reference to codeletion) | 11.321(1.524,84.102) | **0.018** |
| IDH1/2 (reference to wild type) | 0.048(0.015,0.158) | **<0.001** |
| TERT promoter (reference to mutation) | 0.375(0.148,0.951) | **0.039** |
| MGMT promoter (reference to methylated) | 1.455(0.648,3.270) | 0.364 |
| ATRX (reference to intact) | 0.324(0.076,1.381) | 0.128 |
| P53 (reference to positive) | 0.153(0.021,1.136) | 0.066 |

Bold *p* Value < 0.05 is considered statistically significant

**Supplementary table 6** Univariate Cox regression analysis of OS in multiple gliomas patients

| Characteristics | Hazard ratio (95%CI) | *p* Value |
| --- | --- | --- |
| Age | 1.034(1.010,1.059) | **0.006** |
| Sex | 0.724(0.367,1.430) | 0.353 |
| Surgical treatment |  |  |
| Biopsy | 1 | 0.358 |
| non-GTR | 0.395(0.049,3.167) | 0.382 |
| GTR | 0.902(0.323,2.604) | 0.849 |
| Lesion (s) left | 1.516(0.679,3.385) | 0.310 |
| Location |  |  |
| Superficial | 1 | 0.075 |
| Deep | 0.840(0.108,6.513) | 0.867 |
| Both | 2.175(1.076,4.399) | **0.031** |
| Edema |  |  |
| 0mm | 1 | 0.127 |
| 10mm≤ | 0.856(0.114,6.426) | 0.880 |
| >10mm | 1.927(1.005,3.693) | **0.048** |
| Enhancement intensity |  |  |
| None | 1 | **0.014** |
| Mild | 0.775(0.142,4.233) | 0.768 |
| Marked | 3.363(1.187,9.531) | **0.022** |
| WHO grades |  |  |
| Grade1 | - | - |
| Grade2 | 0.210(0.073,0.602) | **0.004** |
| Grade3 | 0.446(0.136,1.463) | 0.183 |
| Grade4 | 1 | **0.009** |
| 1p19q (reference to codeletion) | 5.405(0.716,40.814) | 0.102 |
| IDH1/2 (reference to wild type) | 0.140(0.019,1.023) | 0.053 |
| TERT promoter (reference to mutation) | 0.731(0.310,1.725) | 0.475 |
| MGMT promote  (reference to methylated) | 1.462(0.645,3.312) | 0.363 |
| ATRX (reference to intact) | 1.377(0.520,3.646) | 0.519 |
| P53 (reference to positive) | 1.016(0.353,2.928) | 0.977 |

Bold *p* Value < 0.05 is considered statistically significant

**Supplementary table 7** Clinical and pathological characteristics of 15 cases

| Patient NO. | Type | Age (years) | Sex | Surgical treatment | Survival status | Survival time (months) | WHO grades | Pathology (WHO 2021) |
| --- | --- | --- | --- | --- | --- | --- | --- | --- |
| 1 | multifocal | 38 | male | biopsy | censoring | 0 | 3\4 | Astrocytoma\Astrocytoma |
| 2 | multicentric | 30 | male | non-GTR | alive | 41 | 2\2 | Astrocytoma\Astrocytoma |
| 3 | multicentric | 26 | female | GTR | alive | 42 | 2\2 | Astrocytoma\Astrocytoma |
| 4 | multifocal | 68 | male | Lesion (s) left | censoring | 0 | 4\4 | Glioblastoma\Astrocytoma |
| 5 | multifocal | 51 | male | GTR | died | 15 | 4\4 | Glioblastoma\Glioblastoma |
| 6 | multifocal | 52 | male | GTR | censoring | 0 | 4\4 | Glioblastoma\Glioblastoma |
| 7 | multifocal | 55 | male | GTR | died | 28 | 4\4 | Glioblastoma\Glioblastoma |
| 8 | multifocal | 69 | female | GTR | died | 27 | 4\4 | Glioblastoma\Glioblastoma |
| 9 | multicentric | 30 | male | GTR | alive | 41 | 2\2 | Astrocytoma\Astrocytoma |
| 10 | multicentric | 49 | male | biopsy | dead | 4 | 4\3 | Glioblastoma\Astrocytoma |
| 11 | multicentric | 73 | male | Lesion (s) left | censoring | 0 | 4\4 | Glioblastoma\Glioblastoma |
| 12 | multicentric | 37 | female | Lesion (s) left | censoring | 0 | 4\4 | Glioblastoma\Glioblastoma |
| 13 | multicentric | 57 | male | non-GTR\GTR | alive | 37 | 2\4 | Astrocytoma\Glioblastoma |
| 14 | multifocal | 54 | female | GTR | died | 17 | 4\4 | Glioblastoma\Glioblastoma |
| 15 | multifocal | 58 | male | GTR | censoring | 21 | 3\4 | Astrocytoma\Glioblastoma |
